# Mechanisms of rapid tumor progression after insufficient radiofrequency ablation of hepatocellular carcinoma

**DOI:** 10.3389/fcell.2025.1678304

**Published:** 2025-12-01

**Authors:** Wenming Hou, Ge Ge, Xu Chen, Xinhua Xu

**Affiliations:** 1 Department of Ultrasound, Taizhou Central Hospital (Taizhou University Hospital), Taizhou, Zhejiang, China; 2 Department of Pathology, Third Affiliated Hospital of Zhengzhou University, Zhengzhou, Henan, China; 3 Department of Pathology, The People’s Hospital of Yuhuan, Taizhou, Zhejiang, China

**Keywords:** radiofrequency ablation, hepatocellular carcinoma, recurrence, inflammation, tumor microenvironment

## Abstract

**Background:**

Hepatocellular carcinoma (HCC) is one of the most prevalent malignant tumors globally, characterized by high incidence and mortality rates. Radiofrequency ablation (RFA) is a widely adopted primary ablative therapy for HCC, playing a pivotal role in the management of small HCC and unresectable lesions. However, a subset of HCC patients experiences rapid tumor recurrence and progression following RFA.

**Objective:**

This review aims to summarize the mechanisms underlying rapid tumor progression after RFA for hepatocellular carcinoma, thereby providing a theoretical foundation and future research directions for preventing post-ablation recurrence and progression.

**Methods:**

A systematic review of the literature was performed to synthesize current evidence on the mechanisms of tumor recurrence and progression following RFA for HCC, and to discuss corresponding preventive and therapeutic strategies.

**Results:**

Insufficient radiofrequency ablation (IRFA) is a significant driver of tumor recurrence and progression. Post-ablation relapse is not a simple process of residual cell repopulation but a multifaceted vicious cycle initiated by ablation stress. The core mechanism involves residual tumor cells surviving within a synergistic, pro-tumorigenic microenvironment characterized by thermal injury, hypoxia, inflammation, non-coding RNA dysregulation, epigenetic alterations, and immunosuppression. This hostile niche exerts intense selective pressure, activating a complex molecular cascade that promotes cell survival, immune evasion, and malignant proliferation, ultimately driving rapid progression and invasive recurrence.

**Conclusion:**

IRFA for hepatocellular carcinoma leaves behind residual tumor cells that acquire aggressive malignant phenotypes through diverse biological mechanisms, driving disease recurrence and progression. Enhancing the precision of ablation techniques and developing integrated, multi-modal treatment strategies are promising avenues to suppress post-ablation recurrence and improve long-term patient outcomes.

## 1 Introduction

Hepatocellular carcinoma (HCC) is one of the most prevalent malignant tumors and the leading cause of cancer-related mortality. According to the GLOBOCAN 2020 database, there were 905,677 new cases of HCC in 2020, accounting for 4.7% of all malignant tumors, alongside 830,180 deaths, which represented 8.3% of all malignant tumors, ranking third in mortality after lung and colorectal cancers (Sung et al., 2021). The primary risk factors for HCC include chronic infections with the hepatitis B virus (HBV) and hepatitis C virus (HCV), as well as exposure to aflatoxin-contaminated food and excessive alcohol consumption (Jia L. et al., 2020). In recent decades, HCC incidence has declined in many high-prevalence countries, likely reflecting reduced rates of serum HBV and HCV positivity and decreased aflatoxin exposure within the population (Petrick et al., 2020; Arnold et al., 2020). Despite this decline, the prognosis for HCC remains poor, with incidence rates nearing mortality rates based on population-based surveys (Maluccio and Covey, 2012; Jemal et al., 2011), indicating that the treatment of hepatocellular carcinoma poses significant challenges.

In current clinical practice, hepatectomy, liver transplantation, ablation, transarterial chemoembolization (TACE), and targeted immunotherapy represent the primary treatment modalities for HCC (Chen et al., 2016; Forner et al., 2018; Chakraborty and Sarkar, 2022). Hepatic resection and liver transplantation provide the best prognosis for early-stage HCC; however, due to the asymptomatic nature of early-stage HCC, over 80% of patients diagnosed with HCC present at advanced stages, thereby losing the opportunity for surgical intervention (Brown et al., 2023; Chang et al., 2020). This limitation is compounded by factors such as multicentric tumorigenesis, unresectable tumor locations, and insufficient hepatic functional reserve. Furthermore, the 3- and 5-year recurrence rates in surgically treated patients remain elevated, and the shortage of donors further constrains the application of liver transplantation in HCC cases (Lau and Lai, 2009).

In recent years, local regional therapies for HCC, such as RFA and TACE, have evolved significantly. Currently, RFA is recognized as the standard local ablative therapy for HCC and serves as a viable alternative to surgery, primarily due to its higher local control rates and improved patient survival compared to other local therapies (Nishikawa et al., 2013; Feng et al., 2012). However, some HCC patients experience rapid tumor recurrence and progression following RFA treatment. Increasing evidence indicates that tumors may develop an aggressive phenotype and poor prognosis after RFA, leading to a dramatic deterioration in patients’ conditions (Obara et al., 2008; Kasugai et al., 2007; Ruzzenente et al., 2004). Therefore, elucidating the mechanisms underlying rapid tumor progression after RFA is crucial, as this knowledge may significantly influence the principles and strategies employed in RFA for HCC. A growing body of research suggests that factors such as the inflammatory response, epigenetic modifications of RNA, dysregulation of non-coding RNA expression, autophagy, hypoxia, and tumor microenvironment alterations induced by RFA treatment play pivotal roles in rapid tumor recurrence and progression. Notably, these processes are interrelated; their crosstalk contributes to tumor progression. This review summarizes the roles and mechanisms of HCC in rapid tumor progression following RFA treatment and proposes potential strategies for preventing tumor recurrence and progression after RFA.

## 2 Evidence acquisition and synthesis

This review assessed the latest research articles published in PubMed between 2015 and 2025 and used PubMed to systematically review the original articles. We used the following search strategies: (“Incomplete Ablation” [Title/Abstract] OR “Insufficient Ablation” [Title/Abstract] OR “Residual Tumor” [Title/Abstract] OR “Residual Disease” [Title/Abstract] OR “Ablation Margin” [Title/Abstract] OR “Technical Efficacy” [Title/Abstract] OR “local tumor progression” [Title/Abstract]) AND (“Radiofrequency Ablation” [Mesh] OR “Catheter Ablation” [Mesh] OR “RFA” [Title/Abstract] OR “radiofrequency ablation” [Title/Abstract]) AND (“Carcinoma, Hepatocellular” [Mesh] OR “Liver Neoplasms” [Mesh] OR “HCC” [Title/Abstract] OR “hepatocellular carcinoma” [Title/Abstract] OR “hepatic carcinoma” [Title/Abstract]). 586 manuscripts were retrieved from the literature. We reviewed these manuscripts and prioritized studies that fit our subject and those that are scientifically detailed and well-reported to help us understand them. Finally, 85 manuscripts were selected for our study.

## 3 Overview of RFA

RFA was first reported in 1993 and has since become a widely accepted treatment for HCC. This technique involves the delivery of high-frequency alternating current into the tumor via electrodes, leading to the generation of alternating currents within the tumor that cause ionic churning and, consequently, frictional heat. This process induces coagulative necrosis and tissue desiccation (Lencioni, 2010; Kudo, 2010). Pathologically, the tumor can be classified into three zones following RFA treatment: the central necrotic zone surrounding the electrode, where proteins and tissues rapidly undergo denaturation and necrosis; the peripheral zone, which experiences sublethal hyperthermia; and the outermost layer of tissue that remains unaffected by RFA (Ahmed et al., 2011; Chu and Dupuy, 2014). It has been demonstrated that irreversible cellular damage occurs when tissue is heated to 50 °C for 4–6 min, establishing 50 °C as the low critical temperature for RFA (Mertyna et al., 2007). Although RFA treatment of HCC is predominantly performed using percutaneous access, open surgical and laparoscopic approaches are also viable options (Nishikawa et al., 2013). RFA represents a valuable therapeutic option for patients with unresectable HCC, enhancing the survival rates of those with advanced disease. With ongoing technological advancements, RFA is increasingly being utilized to treat resectable HCC (Tion et al., 2011).

RFA has emerged as the most widely utilized local thermal ablation method, owing to its technical ease of use, safety, satisfactory local tumor control, and minimally invasive nature. RFA has demonstrated excellent efficacy in the treatment of small hepatocellular carcinomas and has achieved comparable outcomes to surgical resection (Chen et al., 2006; Table 1). Compared to hepatic resection, RFA results in less liver damage, thereby leading to a lower incidence of complications (Jin et al., 2020). Additionally, RFA is utilized as a bridging therapy prior to liver transplantation, effectively delaying tumor progression and extending the mean waiting time by more than six to 10 months (DuBay et al., 2011; Brillet et al., 2006; Lu et al., 2005). Furthermore, the combination of RFA with percutaneous ethanol injection, percutaneous hepatic artery chemoembolization, surgical resection, liver transplantation, and targeted molecular therapy has broadened the indications for these approaches and enhanced their respective therapeutic efficacy (Chen et al., 2016; Fe et al., 2014).

**TABLE 1:** Comparison of efficacy between RFA and other therapies for HCC treatment.

Treatment group	Total sample size	Key outcomes	Reference
RFA vs. Stereotactic Body Radiotherapy (SBRT)	166	The SBRT group demonstrated superior outcomes, with 2-year LPFS, median PFS, and 2-year OS rates of 92.7%, 37.6 months, and 97.6%, respectively, compared to 75.8%, 27.6 months, and 93.9% in the RFA group	Xi et al. (2025)
Surgery vs. RFA	302	Surgical resection resulted in a 5-year OS of 74.6% versus 70.4% for RFA. The 5-year RFS rates were 42.9% for surgery and 42.7% for RFA.	Kawaguchi et al. (2025)
RFA vs. RFA combined with Toripalimab	40	The combination therapy group showed improved RFS rates at 6, 12, and 18 months (80%, 62.7%, and 48.7%, respectively) compared to the RFA-alone group (65%, 35%, and 18.8%, respectively)	Wen et al. (2023)
Laparoscopic Hepatectomy vs. RFA	150	Laparoscopic hepatectomy achieved 1-, 3-, and 5-year OS rates of 94.7%, 80.0%, and 74.7%, respectively, versus 93.3%, 78.7%, and 67.9% with RFA. Corresponding RFS rates were 78.7%, 61.3%, and 51.6% for hepatectomy, compared to 69.3%, 53.3%, and 41.0% for RFA.	Song et al. (2024)
TACE combined with RFA vs. Surgery	210	The TACE-RFA group showed 1-, 3-, and 5-year OS rates of 99%, 81%, and 69%, respectively, versus 96%, 81%, and 76% for the surgery group. The 1-, 3-, and 5-year RFS rates were 71%, 38%, and 24% for TACE-RFA, compared to 73%, 43%, and 29% for surgery	Zhang et al. (2025)
Microwave Ablation (MWA) vs. RFA	50	MWA resulted in a longer median DFS (24.5 months) compared to RFA (13.4 months). The 1-, 2-, and 3-year OS rates were 100%, 80%, and 72% for MWA, versus 72%, 64%, and 60% for RFA.	Vogl et al. (2024)
Proton Beam Therapy (PBT) vs. RFA	144	PBT was associated with a higher 2-year LPFS rate (94.8%) compared to RFA (83.9%)	Kim et al. (2021)
Dual-Switching Monopolar (DSM) RFA vs. Conventional Single-Switching Monopolar (SSM) RFA	80	The 2-year LTP rates were 8.5% for DSM-RFA and 4.7% for SSM-RFA. LTP-free survival rates were 90.0% versus 94.4%, and 2-year RFS rates were 54.9% versus 75.7%, respectively	Choi et al. (2021)
Non-Touch (NT) RFA vs. Conventional RFA	73	The NT-RFA group reported significantly lower 1- and 3-year LTP rates (0% and 0%, respectively) compared to the conventional RFA group (15.6% and 24.5%, respectively)	Suh et al. (2021)

LPFS:local progression-free survival, PFS: progression-free survival, OS: overall survival, RFS: recurrence-free survival,LTP:local tumor progression.

RFA is particularly indicated for the treatment of cirrhosis and hepatocellular carcinoma with tumor diameters less than 3 cm (Bruix et al., 2014; Ahn and Kang, 2019), For larger tumors, a single RFA treatment may not suffice to completely encompass the target tumor volume, necessitating multiple overlapping ablation sessions to effectively treat the entire tumor (Kim et al., 2014). However, following RFA treatment, residual tumor tissue often remains in the body, which can limit the treatment’s effectiveness. The reasons for the presence of this residual tumor tissue can be categorized as follows: First, the target ablation temperature may not be adequately achieved due to the ‘heat dissipation’ effect of blood vessels located within or around the tumor. Second, the operator may intentionally lower the temperature to prevent damage to vital organs in proximity to the tumor. Third, particularly in percutaneous procedures, achieving complete overlapping ablation can be operationally challenging (Thanos et al., 2008).

The rapid progression of HCC following residual tumor presence post-radiofrequency ablation treatment has been increasingly documented in the literature. Seki et al. (2001) reported a case of swift tumor progression in a patient with a small HCC measuring 2.5 cm, who underwent RFA and TACE. Although necrosis of the tumor was observed and alpha-fetoprotein (AFP) levels decreased, rapid progression occurred shortly after treatment, resulting in multiple tumors around the treated area. Similarly, another study found that out of 88 HCC lesions, 17 exhibited recurrence post-necrosis, with four patients experiencing rapid intrahepatic tumor progression following RFA treatment (Ruzzenente et al., 2004). Furthermore, numerous studies have highlighted the phenomenon of rapid tumor recurrence after RFA (Su et al., 2021; Zeng et al., 2023; Zhu et al., 2023; Peng et al., 2023; Tang et al., 2023). Moderate hyperthermia during RFA has been shown to enhance the expression of certain cytokines and increase tumor cell activity, thereby accelerating their growth (Markezana et al., 2020). A growing body of evidence indicates that tumor cells persisting after RFA exhibit enhanced malignant biological behaviors, acquired through various mechanisms, which contribute to rapid recurrence and progression following tumor treatment.

## 4 Mechanisms of tumor progression following IRAF

**FIGURE 1:**
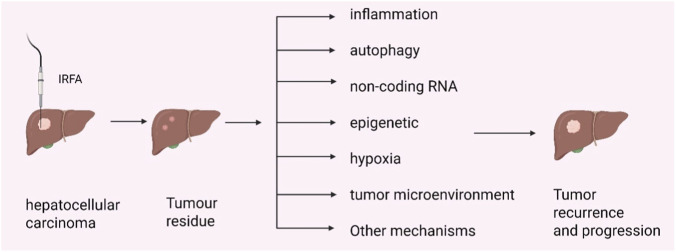
Rapid tumor progression after RFA treatment for HCC. IRFA leaves behind residual tumor cells. Furthermore, the post-ablation state induces a cascade of alterations, including local inflammatory responses, abnormal autophagy, dysregulated non-coding RNA expression, epigenetic modifications, hypoxic stress, and a remodelled tumor microenvironment. These factors collectively confer more aggressive malignant phenotypes upon the residual cells, ultimately driving tumor recurrence and accelerated progression.

The fundamental characteristics of cancer include unlimited proliferation, invasion and metastasis, avoidance of apoptosis, and angiogenesis, all of which are integral to tumorigenesis and progression (Hanahan and Weinberg, 2000; H et al., 2011). Following IRFA, residual tumour cells exhibit enhanced capabilities for proliferation, invasion, metastasis, and epithelial-mesenchymal transition (EMT), coupled with increased angiogenesis. This aggressive phenotype is driven by the activation of an intricate network of signaling pathways that collectively promote tumour recurrence and progression. Key molecular hubs within this network, such as STAT3, NF-κB, and HIF-1α, integrate signals from multiple pathways. For example, HIF-1α activates downstream oncogenic pathways, including TGF-β, Akt, Wnt/β-catenin, and Notch1. Furthermore, STAT3 and NF-κB are frequently activated by inflammatory mediators. Upon activation, these transcription factors translocate to the nucleus and regulate the expression of numerous target genes, thereby exerting diverse pro-tumorigenic effects. In summary, tumour recurrence following IRFA is mediated not by an isolated pathway, but by a complex, interconnected signaling network. The specific mechanisms underlying this process are detailed in the following sections (Figure 1).

### 4.1 The role of inflammation in tumor progression following IRFA

Increasing evidence suggests that inflammation plays a critical role in the rapid progression of tumors. Patients treated with RFA exhibit elevated levels of interleukin-6 (IL-6) and local or systemic inflammatory responses, indicating that the inflammatory response significantly contributes to rapid tumor progression following RFA (Douglas et al., 2008; Chu et al., 2018; Ali et al., 2022; Tan et al., 2018). In the context of IRFA, residual tumor cells at the sub-lethal ablation margins may be influenced both positively and negatively by the inflammatory response (Whiteside, 2008). On one hand, inflammation may enhance tumor cell death through increased neutrophil infiltration and subsequent destruction of tumor cells via an oxidative burst mechanism. Conversely, post-ablative inflammation may lead to the production of growth factors and cytokines by macrophages and lymphocytes, thereby stimulating tumor cell growth within the sub-lethal margin (Balkwill et al., 2005). Several studies have reported elevated levels of the pro-inflammatory factor IL-6 in HCC at the conclusion of RFA treatment (Schälte et al., 2010; Erinjeri et al., 2013). It has been demonstrated in animal models that thermal ablation is associated with peri-ablative inflammation, and elevated levels of IL-6 elicit significant systemic effects, including oncogenic and immunogenic responses (Bulvik et al., 2016). Furthermore, serum from patients undergoing RFA has been shown to significantly enhance the proliferation, migration, and invasion of HepG2 cells, indicating the presence of tumor-promoting factors in the sera of these patients. The expression levels of pro-inflammatory factors such as IL-1β, IL-6, and TNF-α were markedly elevated in these sera, while lymphokines IFN-γ and IL-2 were significantly reduced (Shi et al., 2021). IL-6 can participate in liver regeneration through the NF-kB and STAT3 signaling pathways (Cressman et al., 1995; Cheng et al., 2022; Fazel Modares et al., 2019). IL-6-deficient mice undergoing partial hepatectomy displayed impaired liver regeneration, whereas treatment with IL-6 prior to injury facilitated improved liver regeneration (Cressman et al., 1996). Cytokines, including IL-6, not only promote the regeneration of normal liver parenchyma following injury but also directly support the survival and proliferation of cancer cells (Grivennikov et al., 2010; Li H. et al., 2022; Jin et al., 2018). Several studies have reported that IL-6 can directly influence the proliferation, invasion, and metastasis of cancer cells (Rašková et al., 2022; Che et al., 2019). Given that elevated levels of IL-6 are induced post-RFA treatment, surviving tumor cells at the periphery of the ablation zone may be inadvertently stimulated to proliferate by circulating IL-6, potentially leading to tumor recurrence and rapid progression.

**FIGURE 2:**
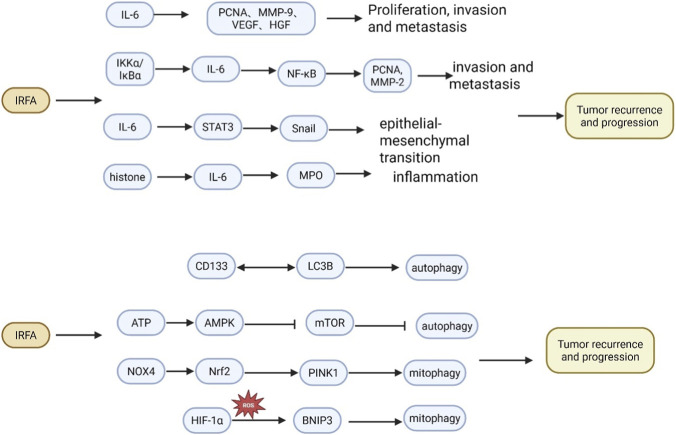
The Role and Mechanism of Inflammation and Autophagy in the Rapid Progression of Tumors Following IRFA Treatment for HCC. IRFA therapy for HCC induces the secretion of inflammatory cytokines, such as IL-6, triggering a subsequent inflammatory response. This response further activates the STAT3 and NF-κB signaling pathways, which in turn promote the proliferation, invasion, and metastatic potential of residual cancer cells, ultimately driving tumor recurrence and progression. Furthermore, following IRFA, surviving tumor cells can undergo autophagy through multiple mechanisms. This process aids their adaptation to the post-ablation stress and facilitates their survival and continued progression.

The promotion of IL-6 release and inflammation following RFA treatment may contribute to the proliferation, migration, and invasion of tumor cells through various mechanisms (Figure 2). Ke et al. reported that insufficient local ablation temperatures may promote tumor proliferation, invasion, and metastasis, with the risk increasing as the target temperature decreases (Ke et al., 2010). Furthermore, the expression levels of proliferating cell nuclear antigen (PCNA), matrix metalloproteinase-9 (MMP-9), vascular endothelial growth factor (VEGF), hepatocyte growth factor (HGF), and IL-6 were significantly elevated in the tumor tissues of the RFA-treated group compared to the control group (Ke et al., 2010). This indicates that IL-6 may promote the expression of PCNA, MMP-9, VEGF, and others following RFA treatment, thereby enhancing proliferation, invasion, and angiogenesis, highlighting its central role in tumor progression. Duan et al. investigated the impact of moderate hyperthermia on the malignant biological behaviors of HCC cells. Their findings indicated that moderate hyperthermia significantly enhanced the proliferation, migration, and invasion of the HCC cell lines HepG2-H and Huh-7-H, which was further corroborated by a marked increase in the expression levels of PCNA and MMP-2 (Duan et al., 2018). Mechanistically, moderate heat treatment elevated the expression of IL-6 and IL-10 in HCC cells. Notably, silencing IL-6 substantially inhibited the proliferation and invasion of HepG2-H and Huh-7-H cells, while also downregulating the expression of PCNA and MMP-2. This suggests that IL-6 mediates the proliferation and invasion induced by moderate hyperthermia. Further studies revealed that the NF-κB pathway was activated following moderate hyperthermia, and the NF-κB inhibitor BAY 11-7082 effectively suppressed IL-6 mRNA expression in HepG2-H and Huh-7-H cells. Additionally, NF-κB was activated in moderate hyperthermia-induced HCC cells via the IKKα/IκBα pathway (Duan et al., 2018). These results imply that moderate hyperthermia activates the NF-κB/IL-6 signaling pathway through IKKα/IκBα, thereby promoting the proliferation, migration, and invasion of hepatocellular carcinoma cells. EMT is a critical mechanism underlying cancer invasion and metastasis. Zhou et al. reported that inadequate heat treatment led to a decrease in E-calmodulin expression and an increase in the expression of vimentin and Snail, resulting in a significant enhancement of EMT in H22 cells and HepG2 cells, as well as a notable increase in IL-6 expression (Zhou et al., 2020). IL-6 activates the Janus kinase (JAK)/STAT3 signaling pathway by binding to its receptor, which induces the phosphorylation of STAT3 at Tyr 705. Phosphorylated STAT3 can translocate to the nucleus to promote the transcription of multiple target genes (Sasser et al., 2007). Previous studies have indicated that increased phosphorylated STAT3 enhances Snail expression, triggers EMT, and promotes tumor progression (Saitoh et al., 2016). Heat treatment of H22 cells also elevated the levels of phosphorylated STAT3. Moreover, combined heat treatment with IL-6 resulted in decreased E-calmodulin expression and significant overexpression of vimentin, Snail, and phosphorylated STAT3, further promoting EMT. Conversely, blockade of the IL-6/STAT3 signaling pathway using AG 490 resulted in decreased STAT3 phosphorylation and reversed the EMT-like changes induced by heat treatment (Zhou et al., 2020). Additionally, in a mouse model of HCC cell *in situ* transplantation, incomplete RFA led to tumorigenic EMT, characterized by reduced E-calmodulin expression and significantly elevated levels of vimentin, Snail, and phosphorylated STAT3 at Y705, corroborating the results of *in vitro* experiments (Zhou et al., 2020). These findings suggest that insufficient RFA enhances EMT through the IL-6/STAT3/Snail pathway, thereby promoting tumor invasion and progression. Circulating histones are recognized as key mediators of both infectious and non-infectious systemic inflammation, exhibiting multiple functions such as inducing endothelial damage, activating immune cells, and promoting cytokine secretion. These activities may elucidate their role in inflammatory injury (Allam et al., 2014; Allam et al., 2013; Abrams et al., 2013). Gu et al. reported an increase in circulating histone levels in patients with HCC following RFA treatment (Gu et al., 2015), Furthermore, after RFA treatment, myeloperoxidase (MPO) activity was significantly enhanced. MPO, a toxic mediator released by neutrophils, serves as a reflection of neutrophil activity. The strong association between histones and MPO supports the hypothesis that histone-triggered neutrophil activation contributes to RFA-associated inflammation (Abrams et al., 2013). Additionally, IL-6 and IL-10 levels were significantly elevated 24 h post-RFA treatment, with a notable correlation between histones and IL-6, suggesting that circulating histones may drive systemic inflammation by promoting cytokine production (Abrams et al., 2013). This study posits that RFA-induced histone release from dead cells may be a key factor in the inflammatory response, offering a novel explanation for the mechanisms underlying RFA-associated systemic inflammatory responses.

In summary, RFA treatment enhances IL-6 secretion and the inflammatory response. IL-6, in turn, can facilitate tumor progression through various mechanisms, including the promotion of inflammatory responses, the upregulation of key proteins involved in tumor proliferation and migration, and the activation of the STAT3 pathway.

### 4.2 The role of autophagy in tumor progression following IRFA

Autophagy is a form of programmed cell death characterized by a self-digestive process wherein cells utilize lysosomes to degrade damaged, denatured, or senescent macromolecules and organelles in response to external environmental factors. Autophagy exhibits a dual role in tumor progression, possessing both pro-tumor and anti-tumor properties. Under normal conditions, autophagy contributes to limiting inflammation and maintaining genomic stability; conversely, it is crucial for tumor survival, providing energy through circulatory mechanisms under metabolic stress. Consequently, autophagy plays a significant role in tumor progression following IRFA treatment of HCC (Figure 2; Chen et al., 2024). Zhao et al. (2018) demonstrated that following RFA treatment in HCC xenograft mice, the proliferation of HCC cells increased, accompanied by a significant upregulation of autophagy markers LC3B, P62, and Beclin-1, indicating autophagy activation. Notably, hydroxychloroquine, an autophagy inhibitor, was employed to suppress the proliferation of HCC cells and induce apoptosis, suggesting that RFA may enhance the proliferation of residual HCC cells via autophagy, thereby facilitating tumor recurrence and progression. Another study revealed that the expression of the hepatocellular carcinoma marker CD133 was significantly elevated in residual HCC cells post-RFA treatment, localized in the autophagosome and positively correlated with LC3B protein expression. Furthermore, the inhibition of autophagy resulted in decreased CD133 expression. Additionally, the knockdown of CD133 inhibited autophagy and suppressed cell viability and invasion in HCC cells after RFA treatment (Wang X. et al., 2018). This suggests that autophagy and CD133 form a positive feedback loop to promote the progression of RFA-treated HCC. mTOR, a well-known negative regulator of autophagy, is modulated by AMPK. Previous studies have shown that heat shock reduces ATP levels, increases the AMP/ATP ratio, and activates AMPK through phosphorylation (Corton et al., 1994). Jiang et al. (2019) confirmed this observation in HCC cells subjected to sublethal heat, where heat stress decreased ATP levels, increased AMPK phosphorylation, and reduced the expression of mTOR and its downstream targets in a time-dependent manner, thereby promoting autophagy activation. Inhibition of AMPK blocked the effects of heat stress-induced AMPK activation and autophagy, suggesting that the ATP-AMPK-mTOR signaling pathway plays a critical role in heat-induced autophagy in HCC cells. Furthermore, with increased macrophage infiltration around the ablation zone and elevated levels of LC3 protein on their surface, macrophages can engulf heat-treated cells through LC3-associated phagocytosis (LAP). This process enhances IL-4-mediated macrophage programming via the PI3Kγ/AKT pathway and inhibits T-cell proliferation, ultimately leading to immunosuppression and promoting residual tumor growth (Liu et al., 2022). Mitophagy, a selective type of autophagy, enhances cell survival by directing dysfunctional mitochondria to autophagosomes for degradation. In response to sublethal heat, the expression of NOX4 is upregulated, leading to the production of mitochondrial reactive oxygen species (ROS). The increased ROS promotes the expression of Nrf2, which subsequently translocates to the nucleus to induce the expression of PINK1. This cascade triggers PINK1-dependent mitophagy, facilitating the elimination of mitochondria damaged by sublethal heat stress and protecting cells from apoptosis (Peng et al., 2023). Xu et al. (2019) demonstrated that IRFA could enhance mitochondrial autophagy via the HIF-1α/BNIP3 pathway, promoting the progression of residual HCC cells *in vitro*. This finding suggests that inhibiting mitochondrial autophagy may help prevent the rapid growth and metastasis of residual HCC following IRFA.

In summary, autophagy is activated following IRFA and facilitates the survival and progression of residual tumor cells. Consequently, pharmacological inhibition of autophagy represents a potential therapeutic strategy to suppress tumor progression post-ablation. However, given the context-dependent dual role of autophagy in tumorigenesis, its precise function in the post-IRFA microenvironment requires further elucidation to validate this therapeutic approach.

### 4.3 Role of non-coding RNAs in tumor progression following IRFA

**FIGURE 3:**
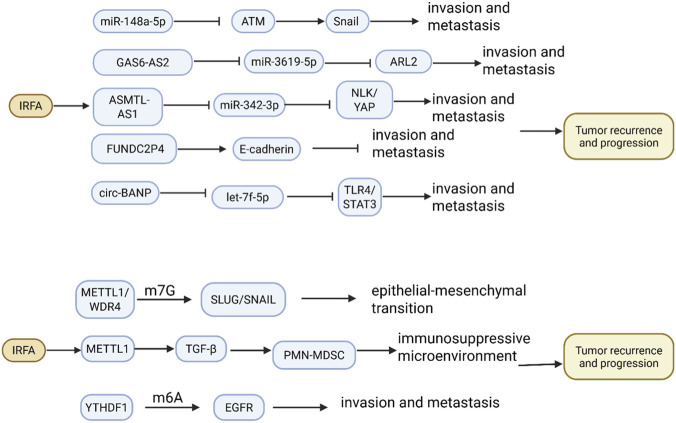
The Role and Mechanism of non-coding RNAs and RNA modification in the Rapid Progression of Tumors Following IRFA Treatment for HCC. IRFA therapy for HCC induces dysregulation in the expression of various non-coding RNAs, including miRNAs, lncRNAs, and circRNAs. This dysregulation disrupts the expression of downstream target genes, which ultimately enhances tumor cell proliferation, invasion, and metastatic capacity, thereby driving tumor recurrence and progression. Furthermore, IRFA promotes specific epigenetic alterations, such as elevated levels of m7G and m6A RNA modifications. These modifications facilitate the activation of downstream oncogenic signaling pathways, further augmenting the invasive and metastatic potential of tumor cells and contributing to disease recurrence.

Non-coding RNAs are a class of RNA molecules that do not encode proteins during transcription; however, they play a crucial role as regulatory factors. These RNAs can interact with downstream genes to form a complex network that regulates gene expression at the post-transcriptional level (Figure 3). Non-coding RNAs can be classified into long non-coding RNAs (lncRNAs), microRNAs (miRNAs), and circular RNAs (circRNAs) (Moran et al., 2012). miRNAs function by degrading or translationally inhibiting target genes through binding to the 3′ untranslated region (3′ UTR) of target mRNAs (Shen et al., 2024). Wang et al. (2020) reported that IRFA can reduce the expression of miR-148a-5p in HCC cells, which subsequently triggers the migration of these cells. Conversely, the overexpression of miR-148a-5p can reverse the migration of HCC cells induced by IRFA. Mechanistically, miR-148a-5p directly targets and reduces the expression of the protein kinase ATM, which in turn increases the protein stability of Snail. lncRNAs also play a significant role in tumor progression following IRFA. Zeng et al. (2018) identified an EMT-associated lncRNA, FUNDC2P4, which was significantly downregulated in HCC cells exposed to sublethal heat. *In vitro* functional assays demonstrated that overexpression of FUNDC2P4 upregulates E-cadherin and inhibits HCC cell proliferation, migration, and invasion. Conversely, silencing FUNDC2P4 downregulated E-cadherin expression and promoted these malignant phenotypes. Conversely, lncRNA GAS6-AS2 was found to be upregulated in HCC cell lines exposed to sublethal heat. Mechanistically, GAS6-AS2 promotes cell survival and invasiveness by sequestering miR-3619-5p, thereby acting as a competing endogenous RNA for ARL2 and enhancing ARL2 expression. In contrast, the inhibition of GAS6-AS2 impeded the proliferation, migration, invasion, epithelial-mesenchymal transition, and stemness of HCC cells (Li et al., 2021). Another study reported the role of lncRNA ASMTL-AS1, an emerging RNA regulator in cancer progression, in residual HCC after IRFA. This study revealed that ASMTL-AS1 was highly expressed in HCC tissues, with its expression further increased after IRFA, correlating with enhanced invasiveness of HCC cells (Ma et al., 2020). Mechanistically, ASMTL-AS1 is transactivated by MYC and promotes NLK expression, thereby activating YAP signaling by sequestering miR-342-3p in HCC. Notably, ASMTL-AS1 can be encapsulated by exosomes and transferred between cells via the NLK/YAP axis, thereby promoting the progression and metastasis of residual tumors after IRFA (Ma et al., 2020). In addition, circRNAs have been implicated in tumor progression following IRFA. Chen J. W. et al. (2022) conducted a study screening circRNAs for differential expression in IRFA-treated HCC tissues, identifying 435 circRNAs that were significantly upregulated and 177 circRNAs that were downregulated. One circRNA, confirmed by Sanger sequencing, was predicted to regulate the expression levels of PD-L1 and VEGFR-1 by acting as a microRNA (miRNA) sponge, thereby forming a circRNA-miRNA-PD-L1/VEGFR-1 regulatory network. Li G. et al. (2022) reported that circ-BANP is upregulated in HCC tissues, with its expression further elevated following IRFA; this elevated expression is correlated with tumor progression. Mechanistically, circ-BANP acts as a competitive endogenous RNA for let-7f-5p, which attenuates the miRNA’s suppression of its target gene, TLR4, and subsequently activates the STAT3 signaling pathway. Furthermore, knockdown of circ-BANP or overexpression of let-7f-5p was shown to inhibit the TLR4/STAT3 signaling pathway, thereby suppressing HCC cell proliferation, migration, invasion, and EMT *in vitro*, as well as tumor growth *in vivo*.

In summary, the dysregulation of non-coding RNA expression following IRFA for HCC is a significant driver of tumor recurrence and rapid progression. The post-ablation microenvironment is characterized by the downregulation of tumor-suppressive non-coding RNAs and the concurrent upregulation of oncogenic non-coding RNAs. Therefore, the targeted modulation of these key non-coding RNAs represents a promising therapeutic strategy to prevent tumor recurrence and inhibit disease progression after IRFA.

### 4.4 Role of RNA modification in tumor progression following IRFA

Current studies have revealed that epigenetic modifications of various RNAs play a crucial role in the rapid cellular response to heat stress. These modifications can significantly influence the progression and recurrence of various tumors by upregulating the expression of key genes (Figure 3). Common RNA modifications include N6-methyladenosine (m6A) methylation and N7-methylguanosine (m7G) methylation. Research has demonstrated that tRNA modifications are critical for tRNA stability and mRNA translation. The m7G modification on tRNA is a highly conserved alteration catalyzed by the METTL1/WDR4 protein complex in mammals. A study indicated that the depletion of the m7G tRNA modification did not affect yeast growth under normal culture conditions; however, yeast lacking the m7G tRNA modification could not survive heat stress, thereby underscoring the critical role of the m7G tRNA modification in the heat response (Alexandrov et al., 2006). Zhu et al. (2023) reported that IRFA significantly upregulated the levels of m7G tRNA modification and its methyltransferase complex component METTL1/WDR4 in various systems, including HCC tissues and cell lines, as well as in HCC patient-derived xenograft mice. Functionally, METTL1-mediated m7G tRNA modification promotes HCC metastasis under sublethal heat exposure, both *in vitro* and *in vivo*. Mechanistically, METTL1 and m7G tRNA modification enhance the translation of SLUG/SNAIL in a codon frequency-dependent manner under sublethal heat stress. These are key genes regulating the EMT program, thereby increasing the invasive and metastatic capacity of HCC cells. Additionally, METTL1 can influence the immunosuppressive tumor microenvironment following IRFA. Zeng et al. (2023) reported that METTL1 expression is upregulated in HCC relapsing after RFA treatment. This upregulation is accompanied by an increase in polymorphonuclear myeloid-derived suppressor cells (PMN-MDSCs) and a decrease in CD8^+^ T cells. Mechanistically, the upregulation of METTL1 enhances the translation of TGF-β2 by inducing myeloid-derived suppressor cells to create an immunosuppressive microenvironment, which significantly affects PMN-MDSC accumulation and subsequently impairs CD8^+^ T cell infiltration. Furthermore, TGF-β significantly attenuates tumor progression induced by IRFA and restores CD8^+^ T cell populations following METTL1 knockdown. These findings suggest that interrupting the METTL1-TGF-β2-PMN-MDSC axis may represent a promising therapeutic strategy to restore anti-tumor immunity and prevent HCC recurrence after RFA treatment.

m6A modification, recognized as the most prevalent RNA epigenetic modification, is catalyzed by a methyltransferase complex that facilitates the m6A modification process, along with RNA-binding proteins that interact with RNA to execute specific functions. This modification plays a crucial role in acute stress responses and cancer progression. A study indicated that elevated levels of m6A modification and the ‘reader’ protein YTH N6-methyladenosine RNA-binding protein 1–3 (YTHDF1) were observed in the transition zone of sublethal heat exposure near the ablation center in an IRFA HCC *in situ* mouse model (Su et al., 2021). Functionally, YTHDF1 enhances HCC cell viability and metastasis. The knockdown of YTHDF1 significantly inhibited tumor metastasis induced by sublethal heat treatment in a mouse model of HCC. Mechanistically, YTHDF1 binds to m6A-modified epidermal growth factor receptor (EGFR) mRNA, thereby enhancing its translation. The combination of YTHDF1 silencing and EGFR inhibition synergistically suppresses HCC cell malignancy. This study suggests that the m6A-YTHDF1-EGFR axis promotes HCC progression following IRFA, and that targeted combined inhibition of m6A modification and EGFR represents a potential strategy for preventing HCC recurrence after RFA treatment.

In summary, IRFA for HCC induces aberrant RNA modifications, notably elevated m7G and m6A levels. These modifications promote the activation of downstream oncogenic signaling pathways, which enhance tumor cell survival, invasion, and metastatic potential.

### 4.5 The role of hypoxia in tumor progression following IRFA

**FIGURE 4:**
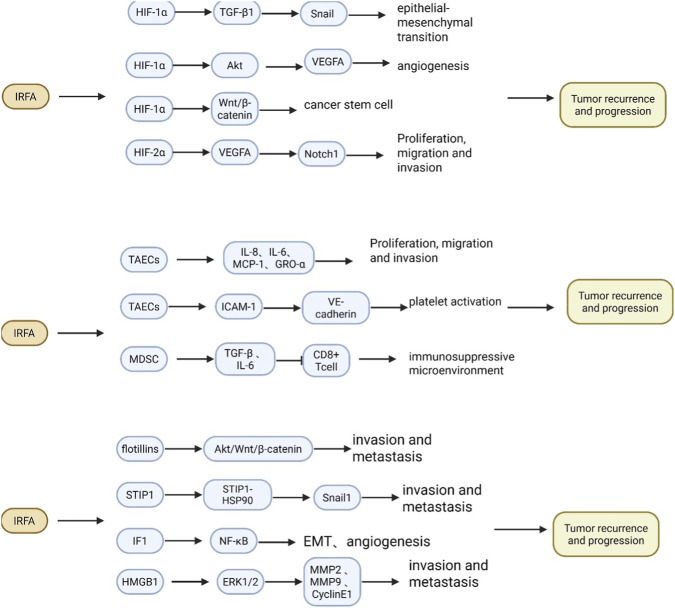
The Role and Mechanism of Hypoxia, Tumor Microenvironment and Other Mechanisms in the Rapid Progression of Tumors Following IRFA Treatment for HCC. Following IRFA for HCC, residual cancer cells are subjected to a hypoxic microenvironment that upregulates the expression of HIFs. This hypoxic response further activates key oncogenic signaling pathways, including TGF-β, Wnt/β-catenin, and Notch1, which collectively promote cancer cell proliferation, invasion, metastasis, and angiogenesis. Concurrently, IRFA remodels the tumor microenvironment by promoting cytokine secretion from TAECs and inducing the upregulation of MDSCs, thereby fostering a potent immunosuppressive niche. Furthermore, IRFA enhances the expression of pro-metastatic proteins such as flotillins, STIP1, IF1, and HMGB1. These proteins facilitate tumor cell invasion and metastasis by activating multiple downstream pathways, ultimately driving tumor recurrence and progression.

It is well established that hypoxia plays a critical role in cancer progression. RFA can induce the formation of a transition zone between normal and necrotic liver tissue, where blood stasis and thrombosis expose residual cancer cells to a hypoxic microenvironment. This environment may significantly contribute to tumor progression following IRFA (Figure 4). Hypoxia-inducible factor (HIF)-1 serves as a key mediator of cellular adaptation to hypoxia, triggering the expression of its target genes. These genes are associated with various cellular processes, including angiogenesis, anticancer drug resistance, and EMT. In a comparative study of HCC patients, Yamada et al. (2014) examined the differences between those undergoing partial hepatectomy without prior RFA and those who had RFA prior to the surgery. The findings indicated that the RFA group exhibited a higher frequency of portal vein infiltration, poorer tumor differentiation, and significantly worse overall and disease-free survival rates compared to the non-RFA group. Additionally, the RFA group demonstrated increased expression of HIF-1 and epithelial cell adhesion molecule (EpCAM) in residual HCC tumors. These results imply that inadequate treatment with RFA may lead to the expression of residual tumors that promote aggressive tumor phenotypes and poor prognoses in recurrent HCC. Some studies have shown that HCC cells subjected to heat treatment and cultured under hypoxic conditions displayed enhanced invasive, metastatic, and chemotherapy-resistant capabilities, as well as a higher percentage of cancer stem cells. However, ameliorating the hypoxic microenvironment or silencing HIF-1α signaling significantly mitigated the malignant behavior of HCC cells. Mechanistic investigations suggest that hypoxia-induced HCC progression may occur through the HIF-1α/TGF-β1/Snail signaling pathway (Tong et al., 2017). Another study found that the expression of VEGFA, HIF-1α, and p-Akt was upregulated in sublethally heat-treated HCC cells, which exhibited stronger pro-angiogenic effects. Inhibition of HIF-1α and silencing of VEGFA diminished cell viability and pro-angiogenic properties, indicating that HIF-1α/VEGFA-induced angiogenesis following interventional RFA may significantly contribute to the rapid growth of residual HCC cells (Kong et al., 2012a). Zhang et al. reported that the upregulation of HIF-1α expression following IRFA promotes the activation of the Wnt/β-catenin signaling pathway, which is essential for increasing invasiveness and acquiring cancer stem cell-like features in HCC cells under hypoxic conditions (Zhang et al., 2023). IRFA also enhances autophagy in residual HCC cells via the HIF-1α/BNIP3 pathway, suggesting a crosstalk between autophagy and hypoxia in tumor progression post-IRFA (Xu et al., 2019). Furthermore, HIF-2α is closely associated with tumor progression, as the mRNA and protein expressions of VEGF, HIF-2α, and Notch1 were significantly elevated in the HCC cell model of IRFA. This elevation correlated with enhanced proliferation, migration, and invasion of HCC cells, while inhibition of HIF-2α reversed these effects (Yang et al., 2022). Mechanistically, HIF-2α promotes HCC proliferation, migration, and invasion through the HIF-2α/VEGF/Notch1 signaling axis, indicating that HIF-2α may serve as a potential target for preventing HCC recurrence and progression following IRFA. In summary, the hypoxic environment and the expression of hypoxia-inducible factors in tumors after IRFA are critical for tumor progression, and strategies aimed at improving the hypoxic environment and inhibiting the expression of hypoxia-inducible factors hold promise for preventing tumor recurrence.

In summary, the establishment of a hypoxic tumor microenvironment and the concomitant upregulation of HIFs following insufficient radiofrequency ablation for HCC are critical drivers of tumor progression. HIFs facilitate this progression by activating key oncogenic signalling pathways, including TGF-β, Wnt/β-catenin, and Notch1, which collectively promote cancer cell proliferation, invasion, metastasis, and angiogenesis. Consequently, therapeutic strategies aimed at alleviating intra-tumoral hypoxia or directly inhibiting HIF signalling represent promising avenues for preventing tumor recurrence.

### 4.6 The role of the tumor microenvironment in tumor progression following IRFA

The tumor microenvironment refers to the non-cancerous cells and components present within a tumor, including the molecules they produce and release. The interaction between tumor cells and the tumor microenvironment plays a decisive role in tumorigenesis, progression, metastasis, and response to therapy (Xiao and Yu, 2021). RFA treatment not only destroys the tumor but also completely remodels the local tissue microenvironment; thus, the tumor microenvironment significantly influences tumor recurrence following RFA (Figure 4). Tumor-associated endothelial cells (TAECs), as a crucial component of the tumor microenvironment, play a key role in angiogenesis, which is essential for tumor growth and invasion. TAECs may enhance the invasiveness of residual HCC through the secretion of cytokines. Kong et al. (2012b) isolated TAECs from HCC and investigated their effects on tumor progression after heat treatment, demonstrating that insufficient RFA promoted the migration and tube formation of TAECs. The expression of E-selectin, intercellular adhesion molecule (ICAM)-1, and vascular cell adhesion molecule (VCAM)-1 on the surface of TAECs was significantly upregulated, increasing their ability to adhere to HCC cells. Significantly elevated levels of IL-8, IL-6, MCP-1, and GRO-α secreted by TAECs may account for their increased invasiveness toward HCC cells. Furthermore, the upregulation of ICAM-1 in TAECs resulted in increased platelet activation and endothelial permeability *in vitro*. The binding interactions between the upregulated ICAM-1 and Ezrin downregulated vascular endothelial (VE)-cadherin expression, while platelet depletion or ICAM-1 inhibition suppressed tumor growth and metastasis following insufficient RFA. These results suggest that ICAM-1 activates platelets and promotes endothelial permeability of TAECs via VE-cadherin after RFA insufficiency (Kong et al., 2021). Type I collagen, one of the most abundant extracellular matrix proteins, is associated with increased invasiveness in various solid tumors, including HCC (Armstrong et al., 2004; Provenzano et al., 2008) It has been reported (Kong et al., 2012a) that increased deposition of type I collagen was observed in the peri-ablative zone following IRFA of HCC in a xenograft model. This collagen deposition activated ERK phosphorylation, thereby enhancing the invasiveness of residual HCC cells after IRFA. In contrast, sorafenib significantly mitigated the pro-tumorigenic effects mediated by type I collagen (Zhang et al., 2018).

The suppressive immune microenvironment established following IRFA is a critical factor in tumor recurrence and progression. Myeloid-derived suppressor cells (MDSCs), which are immature immunosuppressive cells in mice, can be recruited and activated under inflammatory conditions and within the tumor microenvironment (Gabrilovich et al., 2009). MDSCs can directly or indirectly impair T-cell-mediated immune responses, consequently inhibiting the release of various anti-tumor cytokines, such as IFN-γ and TNF-α, and hindering the immune system’s ability to destroy tumors (Marvel and Gabrilovich, 2015). Several studies have shown that the upregulation of MDSCs induced by IRFA in residual tumors is crucial for postoperative recurrence (Zeng et al., 2023; Tang et al., 2023). A significant increase in the percentage of peripheral blood MDSCs and tumor infiltration has been observed following palliative RFA in a mouse tumor model. Furthermore, the expression levels of TGF-β and IL-6 were significantly elevated. After the combined depletion of MDSCs via RFA, levels of Th1 cytokines were higher, which greatly increased the percentage of tumor-infiltrating functional CD8^+^ T cells and inhibited tumor growth (Wu et al., 2020).

In summary, IRFA remodels the tumor microenvironment in HCC by promoting cytokine secretion from TAECs and upregulating MDSCs, thereby fostering an immunosuppressive niche. Reversing this immunosuppression presents a promising therapeutic strategy to prevent tumor recurrence and progression post-ablation.

### 4.7 Role of other mechanisms in tumor progression following IRFA

The mechanism underlying rapid tumor progression following IRFA treatment of HCC is intricate. In addition to the aforementioned mechanisms, numerous signaling pathways influence tumor progression, involving a variety of proteins that ultimately affect the fundamental biological characteristics of tumorigenesis and development, including cell proliferation, migration, invasion, apoptosis, angiogenesis, and EMT (Figure 4). It has been reported that IRFA may promote EMT in HCC cells via the Akt and ERK signaling pathways (Dong et al., 2013). Lipid rafts serve as a physical platform for various molecules involved in biological processes, acting as an organizational center for assembling signaling molecules into functional complexes. Flotillins, comprising two members—flotillin-1 and flotillin-2—are key components of lipid rafts, and their expression has been previously reported to correlate with the progression and poor survival of HCC. In an HCC cell model of IRFA, the expression of flotillin-1 and flotillin-2 was found to be upregulated. Conversely, the inhibition of flotillin-1 or flotillin-2 significantly reduced the inadequate RFA-induced tumor growth *in vitro* and *in vivo*, along with EMT changes and metastasis (Zhang et al., 2019). Mechanistic studies indicate that flotillins activate the Akt/Wnt/β-catenin signaling pathway, thereby altering the EMT status and metastatic potential of heat-treated HCC cells. Stress-induced phosphoprotein 1 (STIP1) is a chaperone molecule that plays a crucial role in maintaining cellular homeostasis in response to heat stress. Knockdown of STIP1 significantly reduced intrahepatic and lung metastasis of HCC in mice following IRFA. Mechanistically, heat exposure induces the formation of the STIP1-Heat Shock Protein 90 (HSP90) complex, which translocates the epithelial transcriptional repressor Snail1 into the nucleus, thereby regulating the transcription of mesenchymal genes (Su et al., 2018). In heat-treated HCC cells, differentially expressed genes were significantly enriched in the MAPK signaling pathway, with c-Met levels markedly elevated in heat-treated HCC cells compared to non-heat-treated counterparts. Inhibition of c-Met activity suppressed the malignant behavior of heat-treated HCC, suggesting that the c-Met/MAPK pathway facilitates the malignant progression of residual HCC cells after IRFA (Jia G. et al., 2020). Additionally, ATPase inhibitory factor 1 (IF1) serves as a poor prognostic predictor, with high expression levels observed in residual tumors post-IRFA. Further investigations revealed that IF1 promotes EMT and angiogenesis via the NF-κB signaling pathway, while also diminishing the sensitivity of HCC cells to sorafenib following IRFA (Kong et al., 2020). Tumor cells that undergo death after IRFA release high-mobility group box 1 (HMGB1), which binds to the receptor for advanced glycosylation end-products, subsequently activating the ERK1/2 pathway and significantly upregulating the expression of MMP2, MMP9, and Cyclin E1, thereby promoting the progression of residual HCC cells (Zhou et al., 2023).

## 5 Potential strategies to prevent tumor recurrence and progression after IRFA treatment

Multiple mechanisms contribute to tumor progression following IRFA, indicating the need for various potential modalities to prevent tumor advancement post-RFA treatment. Inflammation plays a critical role in tumor recurrence after IRFA, and combining RFA with anti-inflammatory treatments presents a promising strategy for mitigating tumor progression. In a study investigating RFA in conjunction with anti-inflammatory treatment for HCC, Jiang et al. implanted two VX2 liver tumors in each rabbit, treating one with RFA. The rabbits were then divided into a control group and three groups receiving high, medium, and low doses of aspirin as anti-inflammatory agents. The study observed that in the high-dose aspirin group, serum levels of IL-6, hs-CRP, and TNF-α were significantly reduced, while tumor volume, as well as lung and kidney metastases, were smaller, and survival time was prolonged compared to the control group. Furthermore, the expression levels of PCNA, MMP-9, and VEGF were significantly decreased, indicating that anti-inflammatory treatment effectively inhibits the proliferation, invasion, and angiogenesis of tumor cells. In conclusion, this study illustrates that inflammation induced by RFA is a significant factor in residual tumor progression, and the anti-inflammatory properties of aspirin can effectively suppress the proliferation, invasion, and metastasis of residual tumor cells, making it a promising candidate for adjuvant therapy following radiofrequency ablation (Jiang et al., 2015).

IRFA-induced upregulation of MDSCs in residual tumors is critical for postoperative recurrence. Several preclinical and clinical studies have confirmed that inhibiting MDSC amplification and activity significantly improves antitumor therapy (Horikawa et al., 2017; Wang P. F. et al., 2018). The depletion of MDSCs through antibody combination resulted in IL-6 levels after day 8 that were not significantly different from pre-treatment levels, alongside a significant increase in the percentage of CD8 T-cells and enhancement of antitumor immunity, ultimately leading to tumor regression (Wu et al., 2020). However, combined MDSC inhibition strategies during IRFA can unexpectedly cause a compensatory increase in PD-L1 expression on residual MDSCs, resulting in immune evasion and subsequent tumor recurrence. To address this issue, a novel size-adjustable hybrid nano-micro-liposome was designed to selectively inhibit MDSCs and block the compensatory upregulation of PD-L1 (Tang et al., 2023). This approach effectively prevented the recurrence of residual tumors in incomplete ablation models following IRFA. Additionally, blockade of cytotoxic T-lymphocyte-associated protein 4 (CTLA-4) inhibited residual tumor progression and improved survival after IRFA in a subcutaneous mouse model of hepatocellular carcinoma (Zhang et al., 2020). Combination therapy utilizing the tumor-lysing peptide LTX-315 alongside an anti-CTLA-4 antibody resulted in improved tumor response and prolonged survival. This strategy also synergistically activated the cGAS-STING pathway and triggered immunogenic cell death, leading to robust antitumor immunity in hepatocellular carcinoma post-IRFA (Sun et al., 2025).

The combination of RFA with recently developed nanomedicines offers a promising approach to remodel the immunosuppressive microenvironment following RFA. For instance, polyethylene glycol (PEG)-modified iron-based single-atom nano-enzymes (P@Fe SAZ) facilitate radiofrequency dynamic therapy (RFDT) using the same trigger, namely, radiofrequency. P@Fe SAZ interacts with the radiofrequency field to generate reactive oxygen species (ROS), enabling low-temperature radiofrequency disintegration discharges (RFDTs) for the release of nanomedicinal drugs. The generated ROS further remodel the immunosuppressive microenvironment enhanced by RFA. Consequently, this nanomedicine-unlocked cryo-RFDT exhibits significant efficacy against residual HCC models post-RFA by inhibiting rapid tumor expansion, suppressing recurrence, prolonging survival, and promoting apoptosis (Fang et al., 2025). Zhu et al. (2024) explored the multifunctional properties of dopamine nanomodulators in combination with GW4869, a neutral sphingomyelinase inhibitor, and amlodipine (AM), an intracellular calcium modulator, to create a tailored delivery platform for precise and tumor-specific release of these therapeutic agents. GW4869 synergistically inhibits exosome biogenesis and secretion alongside AM, which subsequently triggers autophagic degradation of PD-L1. This combination significantly enhances the activation and proliferation of various functional T cell subsets following RFA treatment while effectively reducing the infiltration of immunosuppressive cell types, including regulatory T cells and myeloid-derived suppressor cells. Such favorable remodeling of the tumor microenvironment substantially inhibits the progression and metastasis of HCC after RFA. Chen X. et al. (2022) successfully synthesized biocompatible arsenic-loaded zeolitic imidazolate framework-8 nanoparticles (As@ZIF-8 NPs). These nanoparticles were shown to be more effective than free arsenic trioxide in inhibiting tumor cell proliferation, migration, and invasion under sub-lethal thermal conditions, as well as in inducing EMT and apoptosis *in vitro*. In conclusion, immunotherapy combined with RFA presents a promising strategy for reducing HCC recurrence and progression (Liao et al., 2022).

Hypoxic environment-induced HIF and angiogenesis are critical mechanisms contributing to the recurrence of HCC following irreversible IRFA. Therefore, enhancing the hypoxic environment and inhibiting HIF expression represent potential strategies for mitigating recurrence after IRFA. Sorafenib is recommended for adjuvant treatment in advanced HCC and plays a significant role in managing residual tumors post-IRFA. Xu et al. (2013) reported that in HCC xenografted mice, the expression of HIF-1α and VEGFA was significantly reduced, and tumor growth was markedly slowed in the group treated with RFA combined with sorafenib compared to the RFA-only group. Additionally, an *in vitro* study indicated that sorafenib obstructed the HIF-1α/VEGFA signaling pathway, inhibited tumor invasion, and induced apoptosis in HCC cells (Xu et al., 2014). Furthermore, sorafenib was shown to inhibit the EMT of HCC cells following IRFA and to block the growth and metastasis of HCC cells *in vivo* after IRFA (Dong et al., 2015). In a mouse model of HCC, treatment with melatonin following IRFA resulted in reduced tumor growth and metastasis, a decrease in the proportion of myeloid-derived suppressor cells, and an increase in the proportion of CD8 T-cells, achieving optimal results when combined with anti-programmed death ligand 1 (anti-PD-L1) therapy. Mechanistically, melatonin inhibits EMT, HIF-1α, and PD-L1 expression in tumor cells after IRFA (Ren et al., 2024). This suggests that melatonin can counteract the immunosuppressive tumor microenvironment and enhance the efficacy of immunotherapy by ameliorating hypoxia in residual tumors following IRFA.

Inhibition of autophagy, regulation of non-coding RNA expression, and epigenetic modulation represent potential strategies to prevent tumor recurrence. Furthermore, the combination of additional drugs with IRFA can effectively reduce tumor recurrence and metastasis. For instance, all-trans retinoic acid targets tumor-initiating cells via the PI3K/AKT pathway and significantly inhibits residual tumor growth following IRFA (Wang et al., 2021). The HSP90 inhibitor XL888 enhances apoptosis by down-regulating STAT3 after IRFA (Sun et al., 2021). Metformin effectively blocks the growth of HCC cells *in vivo* after IRFA, demonstrating minimal toxicity in nude mice, and may serve as a preventive measure against the deterioration of HCC post-IRFA (Zhang et al., 2017). Additionally, arsenic trioxide disrupts the paracrine signaling of angiopoietin-1 and angiopoietin-2 by inhibiting p-Akt/Hif-1α, further suppressing angiogenesis in HCC after IRFA (Dong et al., 2022).

## 6 Conclusion and outlook

Rapid tumor progression following RFA poses a significant challenge in the local management of HCC. Extensive research has established that post-RFA recurrence is not merely a process of residual tumor cell regeneration, but a vicious cycle driven by multiple factors activated by ablation stress. The core mechanism involves residual tumor cells, surviving incomplete ablation, being subjected to intense selective pressure within a synergistic microenvironment characterized by thermal injury, hypoxia, inflammation, dysregulated non-coding RNA expression, epigenetic alterations, and immunosuppression. This hostile niche activates a complex cascade of molecular events that intertwine to form a pathogenic continuum—from cellular survival and immune evasion to malignant proliferation—ultimately driving rapid progression and aggressive recurrence. Future research must now pivot from mechanistic exploration to clinical translation. First, achieving complete tumor cell ablation is fundamental, necessitating the development of advanced real-time image-guided technologies and energy-monitoring systems to ensure precise and comprehensive coverage of the ablation zone, thereby physically eliminating the root cause of recurrence. The exploration of novel combination techniques, such as nanomaterial-enhanced irreversible electroporation to boost thermotherapy efficacy, could improve complete ablation rates for irregular or perivascular tumors. Second, to address residual disease, combining RFA with systemic therapies—including anti-inflammatory agents, molecularly targeted drugs, and immune checkpoint inhibitors—represents the most promising direction. The objective should extend beyond eradicating residual cells to actively remodelling the pro-recurrent, immunosuppressive tumor microenvironment, thereby fundamentally blocking the pathways of rapid progression. Finally, within the complex signalling networks, identifying and validating the most critical molecular hubs as optimal therapeutic targets is paramount. In summary, a deeper understanding of the integrated biological mechanisms of post-RFA recurrence, coupled with advances in ablation precision and combination therapies, is the key to preventing this complication and improving long-term survival for HCC patients.
